# Supplementary material for: Dynalign II: common secondary structure prediction for RNA homologs with domain insertions
Source: Nucleic Acids Res. 2014 Nov 21;42(22):13939–48. doi: 10.1093/nar/gku1172 (PMC4267632; doi:10.1093/nar/gku1172)
Supplement: SUPPLEMENTARY DATA [file supp_gku1172_nar-02021-z-2014-File012.zip › manual/javadoc/RNAstructure_java_drawing/source/runners/PlotRunner.html]

PlotRunner


JavaScript is disabled on your browser.


- Overview
- Package
- Class
- Tree
- Index
- Help

- Prev Class
- Next Class

- Frames
- No Frames

- All Classes

- Summary:
- Nested |
- Field |
- Constr |
- Method

- Detail:
- Field |
- Constr |
- Method


RNAstructure\_java\_drawing.source.runners

## Class PlotRunner

- java.lang.Object
- - RNAstructure\_java\_drawing.source.runners.RunnerTemplate
  - - RNAstructure\_java\_drawing.source.runners.PlotRunner

- All Implemented Interfaces:
  :   Serializable

  ---

    

  ```
  public class PlotRunner
  extends RunnerTemplate
  implements Serializable
  ```

  A class that runs RNAstructure Java dot plot drawing independently of the
  main RNAstructure application.

  See Also:
  :   Serialized Form

- - ### Field Summary

    Fields

    | Modifier and Type | Field and Description |
    | --- | --- |
    | `private static long` | `serialVersionUID` |
  - ### Constructor Summary

    Constructors

    Constructor and Description

    `PlotRunner()`

    Constructor.
  - ### Method Summary

    Methods

    | Modifier and Type | Method and Description |
    | --- | --- |
    | `static void` | `main(String[] args)` The main method. |
    | `protected String` | `selectFile()` Select an image file. |

    - ### Methods inherited from class RNAstructure\_java\_drawing.source.runners.RunnerTemplate

      `addClosingListener`
    - ### Methods inherited from class java.lang.Object

      `clone, equals, finalize, getClass, hashCode, notify, notifyAll, toString, wait, wait, wait`

- - ### Field Detail


    - #### serialVersionUID

      ```
      private static final long serialVersionUID
      ```

      See Also:
      :   Constant Field Values
  - ### Constructor Detail


    - #### PlotRunner

      ```
      public PlotRunner()
      ```

      Constructor.
  - ### Method Detail


    - #### main

      ```
      public static void main(String[] args)
      ```

      The main method.

      Parameters:
      :   `args` - The command line arguments (ignored).


    - #### selectFile

      ```
      protected String selectFile()
      ```

      **Description copied from class: `RunnerTemplate`**

      Select an image file.

      **Specified by:**
      :   `selectFile` in class `RunnerTemplate`

      Returns:
      :   The image file.


- Overview
- Package
- Class
- Tree
- Index
- Help

- Prev Class
- Next Class

- Frames
- No Frames

- All Classes

- Summary:
- Nested |
- Field |
- Constr |
- Method

- Detail:
- Field |
- Constr |
- Method
